# Multiparameter neuroimaging study of neurovascular coupling changes in patients with end‐stage renal disease

**DOI:** 10.1002/brb3.3598

**Published:** 2024-06-24

**Authors:** Wei Sun, Chen Li, Zhuqing Jiao, Tongqiang Liu, Haifeng Shi

**Affiliations:** ^1^ Department of Radiology The Affiliated Changzhou Second People's Hospital of Nanjing Medical University, Changzhou Second People's Hospital, Changzhou Medical Center, Nanjing Medical University Changzhou China; ^2^ Graduate College, Dalian Medical University Dalian China; ^3^ Department of Nephrology The Affiliated Changzhou Second People's Hospital of Nanjing Medical University, Changzhou Second People's Hospital, Changzhou Medical Center, Nanjing Medical University Changzhou China; ^4^ School of Computer Science and Artificial Intelligence Changzhou University Changzhou Jiangsu China

**Keywords:** arterial spin labeling, cognitive impairment, end‐stage renal disease, functional magnetic resonance imaging, neurovascular coupling

## Abstract

**Purpose:**

To assess changes in neurovascular coupling (NVC) by evaluating the relationship between cerebral perfusion and brain connectivity in patients with end‐stage renal disease (ESRD) undergoing hemodialysis versus in healthy control participants. And by exploring brain regions with abnormal NVC associated with cognitive deficits in patients, we aim to provide new insights into potential preventive and therapeutic interventions.

**Materials and methods:**

A total of 45 patients and 40 matched healthy controls were prospectively enrolled in our study. Montreal Cognitive Assessment (MoCA) was used to assess cognitive function. Arterial spin labeling (ASL) was used to calculate cerebral blood flow (CBF), and graph theory–based analysis of results from resting‐state functional magnetic resonance imaging (rs‐fMRI) was used to calculate brain network topological parameters (node betweenness centrality [BC], node efficiency [Ne], and node degree centrality [DC]). Three NVC biomarkers (CBF‐BC, CBF‐Ne, and CBF‐DC coefficients) at the whole brain level and 3 NVC biomarkers (CBF/BC, CBF/Ne, and CBF/DC ratios) at the local brain region level were used to assess NVC. Mann–Whitney *U* tests were used to compare the intergroup differences in NVC parameters. Spearman's correlation analysis was used to evaluate the relationship among NVC dysfunctional pattern, cognitive impairment, and clinical characteristics multiple comparisons were corrected using a voxel‐wise false‐discovery rate (FDR) method (*p* < .05).

**Results:**

Patients showed significantly reduced global coupling coefficients for CBF‐Ne (*p* = .023) and CBF‐BC (*p* = .035) compared to healthy controls. Coupling ratios at the local brain region level were significantly higher in patients in 33 brain regions (all *p* values < .05). Coupling ratio changes alone or accompanied by changes in CBF, node properties, or both CBF and node properties were identified. In patients, negative correlations were seen between coupling ratios and MoCA scores in many brain regions, including the left dorsolateral superior frontal gyrus, the bilateral median cingulate and paracingulate gyri, and the right superior parietal gyrus. The correlations remained even after adjusting for hemoglobin and hematocrit levels.

**Conclusion:**

Disrupted NVC may be one mechanism underlying cognitive impairment in dialysis patients.

## Graphical abstract

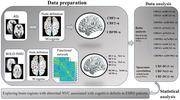

## 1 INTRODUCTION

### 1.1 Background of end‐stage renal disease (ESRD)

ESRD is the final stage of chronic kidney disease. Patients with ESRD have a glomerular filtration rate of < 15 mL/min/1.73 m^2^, or a reliable renal capacity of < 10%, with no or almost no renal function. Such patients require continuous renal substitution treatment. This has become a worldwide public health problem (Webster et al., 2017), especially as epidemiological studies reveal cognitive dysfunction in the majority of patients with end‐stage renal disease. The mechanism for the development of cognitive dysfunction in ESRD patients is unclear, and possible mechanisms include metabolic abnormalities associated with renal failure, vascular lesions, etc. Factors associated with renal failure may include uremic toxins, anemia, etc. (Cheng et al., 2019). There are also many dialysis‐related risk factors, including duration of dialysis and uremic toxins, which are cumulative (Olczyk et al., 2022). These risk factors contribute to cognitive decline in ESRD patients. Cognitive impairment in turn can complicate the treatment of ESRD and thus lead to poor clinical outcomes such as dialysis withdrawal, hospitalization, or death (Pépin et al., 2021). Understanding the effects of hemodialysis treatment on cognitive function may therefore allow for early intervention and improved prognosis in patients with ESRD.

### 1.2 Neuroimaging techniques in ESRD research

Neuroimaging is one technique that can be used to investigate the neuropathological mechanisms underlying cognitive impairment in patients with ESRD (Peng et al., 2021). Arterial spin labeling (ASL) imaging, for instance, is a simple, noninvasive neuroimaging method that is often used to evaluate cerebral perfusion. During ASL acquisition, multiple pairs of labeled and control images are acquired and the CBF is averaged to generate an absolute CBF map and a CBF visualization map. In most previous studies, ESRD patients showed altered CBF by ASL imaging (Chai et al., 2021; Li et al., 2024; Wang et al., 2023). Abnormal brain activity in regions related to cognitive function was found using blood oxygenation level‐dependent (BOLD) resting‐state functional magnetic resonance imaging (rs‐fMRI) (Buckner et al., 2008; Cao, Zhang, & Liu, 2022; van den Heuvel, 2010). Interpretation of neuroimaging results requires careful analysis. Graph theory, which relies on quantitative analysis, can be used to visualize the complex whole brain network by analyzing the network comprehensively in terms of local separation, global integration, and network analysis (Wu et al., 2020).

### 1.3 NVC and cognitive function in ESRD

To date, most neuroimaging studies in patients with ESRD have focused on the relationship between cognitive impairment and abnormal intrinsic neural activity or abnormal CBF (Cheng et al., 2019; Ni et al., 2014). NVC is a mechanism of the neurovascular unit (NVU) that regulates CBF to meet the energy demands of the neuronal activity and maintain balance (Phillips et al., 2016). NVU damage was proven to cause neurological dysfunction, especially when vascular integrity and cerebral autoregulation were impaired (Schaeffer & Iadecola, 2021; Zhu et al., 2022). Based on the NVC hypothesis, brain regions with stronger connectivity tend to have higher spontaneous neuronal activity with greater metabolic demand, resulting in increased perfusion (Kuschinsky, 1991; Venkat, Chopp, & Chen, 2016). Moreover, several studies have identified an association between CBF and brain connectivity. For example, network analyses have found correlations between CBF and anatomical and functional connections (Liang, Connelly, & Calamante, 2014; Liang, Zou, He, & Yang, 2013); and ICA has shown correlations between CBF and connectivity in several resting brain networks (Jann et al., 2015). Liang and colleagues have proved the tight coupling between CBF and FC strength (FCS) in the normal brain, where regions with high connectivity degree exhibited high CBF (Liang, Zou, He, & Yang, 2013), supporting the NVC hypothesis that dense FC tends to be accompanied by increasing blood perfusion (Zhu et al., 2017). Thus, the spatial correlation between CBF and a measure of local brain connectivity has been proposed as a surrogate index for NVC. Several studies have assessed NVC using two different indices, namely, the correlation between ASL and resting‐state functional magnetic resonance imaging (rs‐fMRI) at the global level and the ratio of perfusion and neural activity or brain connectivity at the regional level (Li et al., 2022; Li et al., 2023; Liang et al., 2013; Shang et al., 2022; Zhu et al., 2017). For example in a recent study, they investigated the four types of NVC between amplitude of low‐frequency fluctuation (ALFF), fractional ALFF, regional homogeneity, degree centrality, and CBF, and indicated that ESRD patients showed NVC dysfunction in global gray matter and multiple brain regions due to the mismatch between CBF and neural activity (Hu et al., 2024). Reduced or disrupted NVC has been shown to be a major cause of cognitive dysfunction in numerous pathological conditions such as Alzheimer's disease, schizophrenia, diabetes, hypoxia, stroke, and traumatic brain injury (Barloese et al., 2022; Chen et al., 2023; Hinzman et al., 2014; Rossetti et al., 2021; Salinet et al., 2019; Solis, Hascup, & Hascup, 2020; Zhu et al., 2017). However, few studies have assessed whether such changes in NVC are related to changes in cognitive function among patients with ESRD (Jin et al., 2020). In this study, nodal parameters based on graph‐theoretic analysis are important indicators to represent brain connectivity in local brain regions. Node betweenness centrality (BC) reflects the contribution of each node to the shortest path between all other nodes, the higher the BC value, the greater the influence on the network circulation or connectivity. Node efficiency (Ne) reflects the communication ability of a single node in the network, with high Ne indicating that the node has a strong transmission ability. Node degree centrality (DC) is defined as the number of nodes or edges directly connected to node “I” and is commonly used to measure the importance of a single node (He, Chen, & Evans, 2007; Salvador et al., 2005). The CBF‐nodal parameter correlation represents the consistency of spatial distribution between blood supply and functional hubs, the CBF/nodal parameter ratio represents the cerebral blood supply per unit of connectivity hub, reflecting the NVC. We sought to explore changes in coupling coefficients and coupling ratios among patients with ESRD using a combination of rs‐fMRI based on graph theory analysis and ASL imaging, we aimed to compare the differences in NVC between patients and healthy controls to determine whether these changes were associated with alterations in cognitive function.

## 2 MATERIALS AND METHODS

### 2.1 Participants

This study was approved by the Ethics Committee of the Affiliated Changzhou No. 2 People's Hospital of Nanjing Medical University (Number: KY039‐01). All participants provided informed consent to participate in the study. Patients who underwent hemodialysis at the Affiliated Changzhou No. 2 People's Hospital of Nanjing Medical University Hospital between February 2022 and December 2022 were eligible for study inclusion if they had been diagnosed with ESRD based on the Kidney Disease Quality of Life Guideline classification; had been undergoing regular maintenance hemodialysis for > 3 months (3 times/week at the dialysis center); had a Montreal Cognitive Assessment (MoCA) score < 26; and were aged between 30 and 65 years. Patients were excluded from the analysis if they had a history of other neuropsychiatric disorders such as epilepsy, dementia, brain injury, or brain tumor; had undergone a renal transplant; had an acute infection; had a contraindication to MRI, such as claustrophobia or the presence of metal implants; or had demonstrated substantial head motion artifact on imaging.

Healthy participants enrolled from the local community were assigned to the healthy control group. Healthy participants were required to have a MoCA score > 26 and an absence of known renal disease or other systemic diseases. Exclusion criteria for healthy participants were the same as those used for study patients. Healthy participants were matched to patients based on age, sex, and years of education.

All patients and healthy participants were right‐handed and were fully capable of completing neuropsychological testing.

Of 103 initial participants (52 patients with ESRD undergoing dialysis and 51 healthy controls), 18 were excluded (1 patient who had extensive focus of encephalomalacia in the left frontal lobe, 1 healthy control who had left middle cerebral artery occlusion, 6 patients and 1 healthy control who demonstrated head motion > 3.0 mm, and 9 healthy controls who had MoCA scores < 26). The final study population therefore consisted of 45 ESRD patients (22 males and 23 females, mean age 49 ± 11 years) undergoing dialysis and 40 healthy controls (19 males and 21 females, mean age 46 ± 10 years). The main cause of ESRD in study patients was glomerulonephritis (31 cases). The remaining cases were caused by immunoglobulin A (IgA) nephropathy (8 cases) or membranous nephropathy (6 cases).

### 2.2 Clinical characteristics

Demographic and clinical data were collected from the medical records of patients with ESRD. These tests included assessment of white and red blood cell counts; hemoglobin and hematocrit values; and levels of fasting glucose, urea nitrogen, creatinine, uric acid, cholesterol, triglycerides, and calcium. No laboratory tests were performed in healthy control participants.

### 2.3 Neuropsychological assessment

MoCA was administered by a trained clinical psychologist (Z.A.A., 3 years of experience) 2 h before the participant's MRI examination, with a prescribed test time of ≤10 min. MoCA was used as a screening tool for global cognitive function, including visuospatial skills, attention, memory, orientation, language, executive function, conceptual thinking, and computation, with patients scoring < 26 classified as cognitively impaired (Davis et al., 2015).

### 2.4 MRI data acquisition

The selected hemodialysis patients in this study underwent MRI examination at one day after dialysis. Because patients undergoing hemodialysis experience much interdialytic hemodynamic changes and fluctuations in body fluid content, several studies have confirmed that fluctuation in CBF during the interdialytic cycle can normalize at the end of the session (Metry et al., 2002; Prohovnik et al., 2007), so the effect of hemodynamic fluctuation may be not a significant confounding factor. MRI data were acquired using a 3.0T scanner (Discovery MR750W; General Electric Medical Systems; Milwaukee, WI, USA) equipped with a standard 32‐channel head and spine combined coil. Participants were provided with earplugs and were then placed in a supine position, with foam pads used to minimize head movement. All subjects were instructed to keep their eyes closed, relax, move as little as possible, think of nothing in particular, and stay awake during the scans. Throughout the scanning process, we monitored if they opened their eyes or moved their heads through a monitor. At the end of the scan, we asked them if they remained awake and verified their cooperation with us. Conventional sequence scans, including T1‐weighted, T2‐weighted, and T2 fluid attenuated inversion recovery (FLAIR) sequences, were performed first, and the results were read by 2 blinded diagnostic physicians to rule out the presence of intracerebral lesions (J.A.A., 4 years of experience, and S.A.A., 13 years of experience). The rs‐fMRI images were then acquired using a gradient‐recalled echo‐planar imaging (GRE‐EPI) sequence. CBF was assessed using a pseudocontinuous ASL sequence with background suppression, and a 3‐dimensional brain volumetric imaging (3D‐BRAVO) sequence was used to acquire high‐resolution T1‐weighted structural images of the whole brain for postprocessing functional image alignment.

### 2.5 Scan parameters

The parameters for the GRE‐EPI sequence were as follows: repetition time (TR) = 2000 ms; echo time (TE) = 40 ms; slice thickness = 4.0 mm; flip angle (FA) = 90°; field of view (FOV) = 240 mm × 240 mm; and matrix = 64 × 64. The scanning time was 8 min.

The parameters for the pseudocontinuous ASL sequence were as follows: TR = 5335 ms; TE = 10.7 ms; slice thickness = 4.0 mm; post label delay (PLD) = 2525 ms; FOV = 240 mm × 240 mm; and number of scanned slices = 36. The scanning time was 3 min 44 s.

The parameters for the 3D‐BRAVO sequence were as follows: TR = 7.5 ms; TE = 2.5 ms; inversion time = 450 ms; layer interval = 1.0 mm; FA = 15°; FOV = 240 mm × 240 mm; slice thickness = 1 mm; and number of scanned layers = 154. The scanning time was 3 min 51 s.

### 2.6 Image processing

#### 2.6.1 ASL imaging

CBF images were generated automatically by the GE MRI scanner. MATLAB_2018b platform (https://www.mathworks.com/), SPM12 (Statistical parametric mapping, http://www.fil.ion.ucl.ac.uk/spm/), and REST v1.8 (Resting‐State fMRI Data Analysis Toolkit, http://www.restfmri.net/forum/) were used to preprocess the CBF data. The preprocessing procedure included (1) alignment of 3D‐ASL and 3D‐BRAVO images using SPM's Coregister tool; (2) segmentation of the aligned high‐resolution brain structure images using SPM's Segment tool to identify the gray matter, white matter, and cerebrospinal fluid; (3) spatial normalization of the CBF maps using SPM's Normalise tool to convert the CBF maps to the standard brain space of Montreal Neurological Institute (MNI) and to generate images with the same orientation and size (thus eliminating differences related to the different anatomies of each participant); (4) spatial smoothing of normalized images with a full width at half maximum (FWHM) of 5 mm × 5 mm × 5 mm using SPM's Smooth tool to improve the signal‐to‐noise ratio of the images; and (5) extraction of the CBF values of 90 brain regions within the automated anatomical labeling (AAL) template using the REST toolbox. Absolute CBF is expressed in units of 100 grams per minute per milliliter.

#### 2.6.2 rs‐fMRI

MATLAB 2018b, SPM12, and DPARSF_V5.1_201001 (Data Processing Assistant for Resting‐State, http://www.rfmri.org/DPARSF) were used to perform preprocessing operations on the rs‐fMRI data. The steps were as follows: (1) The first 10 images were discarded to allow for steady‐state longitudinal magnetization. (2) The remaining data from 230 timepoints were analyzed for slice timing and head motion correction (participant data were excluded if head movement was > 3 mm or 3°). (3) Spatial normalization to the MNI template was performed. (4) Images were warped into the standard stereotaxic space at 3 mm × 3 mm × 3 mm of the standard MNI. (5) Spatial smoothing was performed with a 6‐mm full width at half‐maximum Gaussian kernel. (6) Blood oxygen level‐dependent (BOLD) signal in the range of 0.01–0.08 Hz was selected as the filtering bandwidth to filter out the physiological noise signal from the high‐frequency band and to screen out the signal drift generated by the low frequency. Finally, the nuisance covariates, including the 6 head motion parameters as well as average signals from cerebrospinal fluid and white matter, were removed by linear regression.

Once preprocessing was complete, the MATLAB‐based GRETNA (Graph Theoretical Network Analysis) 2.0 (https://www.nitrc.org/projects/gretna) toolbox was used to construct the functional brain network and to analyze the topological properties of the network (Wang et al., 2015). To ensure reproducibility of the experiment, the brain was partitioned into 90 regions using the AAL template, and each brain region was defined as a node. The average time series between any nodes was extracted and Pearson's correlation analysis was performed between two, that is, the construction of edges. For each participant, a 90 × 90 functional network binarization matrix was generated at the individual level. Fisher's Z‐transformation was then performed to generate a Z‐matrix, and with matrix sparsity used as the threshold, an undirected binary matrix with a matrix sparsity range of 0.1 to 0.5 and a step size of 0.01 was constructed (the small‐world values of all participants were checked more than once to avoid selecting a threshold range that was too wide to produce connected nodes and networks with small‐world characteristics) (Wu et al., 2020). For each sparsity threshold, the topological property parameters of the brain functional network were calculated. Node properties included DC, Ne, and BC. Areas under the curve (AUCs) of the functional network topological properties were then calculated within the entire sparsity threshold to provide scalars that did not depend on a specific threshold selection (Zhang et al., 2011).

### 2.7 NVC analysis

#### 2.7.1 Whole brain level NVC analysis

**FIGURE 1:**
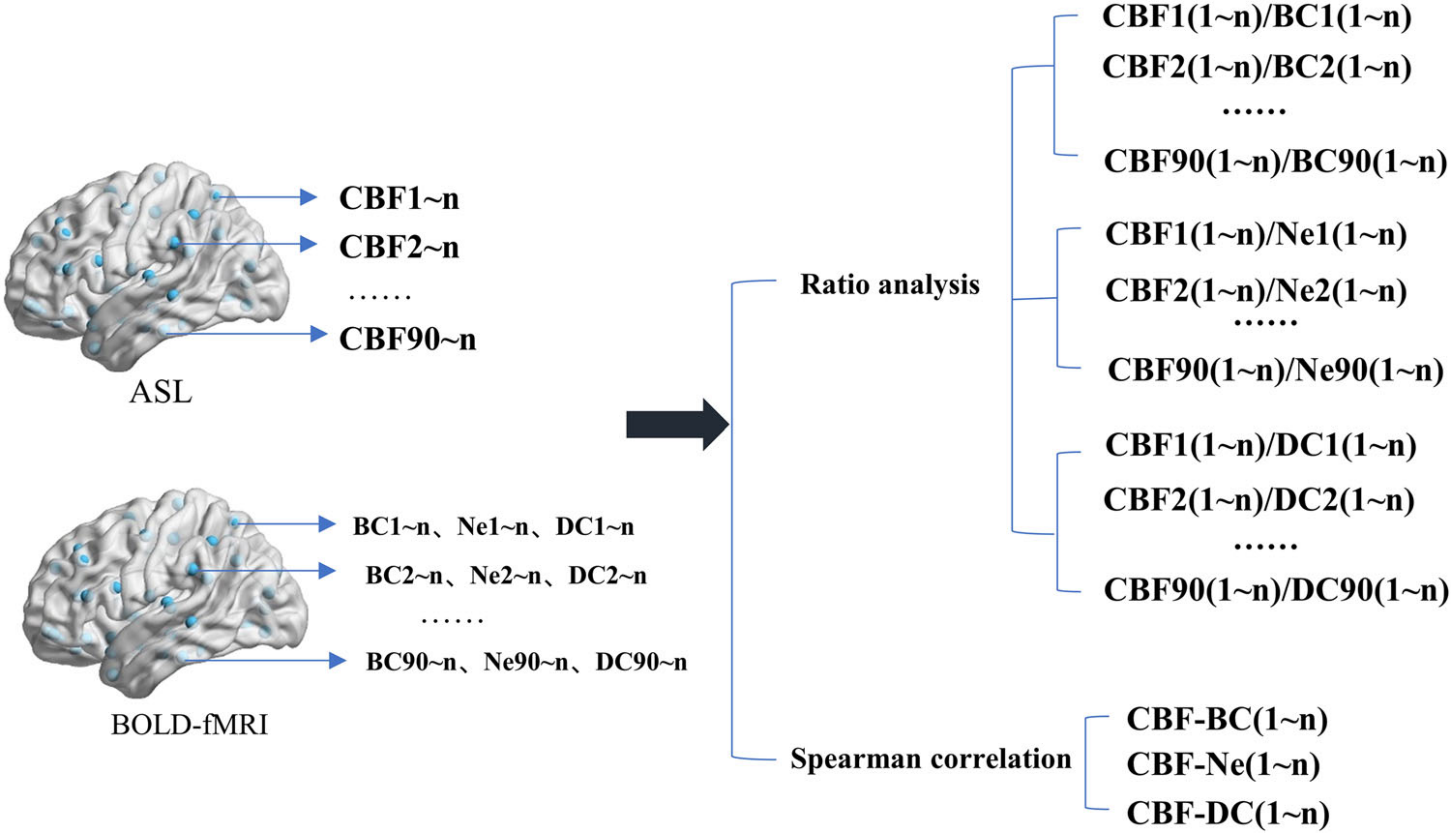
Process for neurovascular coupling analysis (whole brain level and local brain level). The *n* represents the nth participant. CBF, cerebral blood flow; ASL, arterial spin labeling; BOLD, blood oxygen level dependent; fMRI, functional magnetic resonance imaging; BC, betweenness centrality; Ne, node efficiency; DC, degree centrality.

An overall Spearman's correlation analysis was performed between brain perfusion images (CBF) and neuronal activity images (BC, Ne, and DC) using SPSS software (SPSS version 23.0; SPSS, Chicago, Ill, USA). For each participant, 3 NVC coefficients (CBF‐BC, CBF‐Ne, and CBF‐DC) were evaluated at the whole brain level. The NVC coefficients represent global NVC and reflects the coordination between the brain connectivity and the blood supply (Figure 1).

#### 2.7.2 Local brain region level NVC analysis

We computed the regional CBF/BC, CBF/Ne, and CBF/DC ratios for each brain region. For each participant, 3 × 90 NVC ratios were evaluated. The ratio represents the regional NVC across the brain and reflects a balance between CBF and brain connectivity. Regions with higher values tend to have more metabolic demands as they communicate with the rest of the brain (Liang et al., 2013) (Figure 1).

### 2.8 Statistical analysis

Imaging parameters, demographic data, clinical data, and neuropsychological test scores were analyzed using SPSS (version 23.0). Measures that conformed to a normal distribution were expressed as mean ± standard deviation. Median and upper and lower quartiles were used for measures that did not conform to a normal distribution. A chi‐square test was used to compare sex‐based differences between patient and control groups. A Mann–Whitney *U* test was used to detect between‐group differences in CBF and NVC parameters. The remaining parameters were assessed using a two‐sample independent Student *t*‐test. A *p* value < .05 was used to indicate statistical significance. Multiple comparisons were corrected in R Studio (version 3.6.3) using the false‐discovery rate (FDR) criterion‐corrected *p* < .05.

If the indicators of both groups conformed to a normal distribution, Pearson's correlation analysis was performed; if they did not conform to a normal distribution, Spearman's correlation analysis was used. Spearman's correlation analysis was used to calculate NVC coefficients at the individual level as well as to study the relationship between MoCA scores and changes in NVC parameters or clinical indicators in hemodialysis patients at the group level. Given that anemia may be a predominant risk factor for neurocognitive impairment, partial correlation analysis was further performed with adjustment of the hemoglobin and hematocrit levels. A *p* value < .05 (uncorrected) was again used to indicate statistical significance.

### 2.9 Driving diagram and overlapping diagram

To better determine what may drive differences in NVC ratios, intergroup difference maps for CBF, nodal parameters, and CBF/nodal parameter ratios were projected onto a driving diagram. An overlapping diagram of the brain regions with changes in CBF, nodal parameters, and NVC ratio parameters was also produced to represent the sensitivity of each index (Figure 3).

## 3 RESULTS

### 3.1 Demographic, clinical, and neuropsychological results

No significant differences were observed between the 2 groups in terms of age (*p* = .161), sex (*p* = .898), or years of education (*p* = .913). MoCA scores were significantly lower in patients with ESRD than in healthy controls (*p* = .003). According to the normal range of these indicators in a healthy population. Our patients had low levels of red blood cells count, hemoglobin, hematocrit value, and calcium. The levels of urea nitrogen, creatinine, and uric acid are elevated (Table 1).

**TABLE 1:** Demographic and clinical characteristics.

Variable	Patients with ESRD (*n* = 45)	Healthy controls (*n* = 40)	Statistical test	*p* Value
Age, years	49 ± 11	46 ± 10	*t* = 1.414	.161
Male, *n* (%)	22 (49)	19 (48)	χ^2^ = 0.016	.898
Education, years	9.5 ± 2.7	9.8 ± 2.5	*t* = −1.11	.913
MoCA score	22.6 ± 3.6	28.1 ± 2.2	*t* = −3.05	.003
Laboratory data				
White blood cell count, 10^9^/L	6.54 ± 0.35			
Red blood cell count, 10^12^/L	3.19 ± 0.14			
Hemoglobin, g/L	95.36 ± 4.12			
Hematocrit, %	27.17 ± 1.79			
Platelets, 10^9^/L	186.39 ± 11.31			
Fasting glucose, mmol/L	5.25 ± 0.32			
Urea nitrogen, mmol/L	20.16 ± 1.54			
Creatinine, μmol/L	892.17 ± 79.21			
Uric acid, μmol/L	386.66 ± 20.51			
Cholesterol, mmol/L	4.04 ± 0.22			
Triglycerides, mmol/L	1.72 ± 0.24			
Calcium, mmol/L	2.11 ± 0.05			

*Significantly difference. Unless otherwise noted, data are mean ± standard deviation.

ESRD, end‐stage renal disease; *t*, two‐sample *t*‐test; χ^2^, chi‐square test; MoCA, Montreal Cognitive Assessment.

### 3.2 Group differences in CBF and network topological properties

Once FDR correction was performed, patients with ESRD were found to have significantly higher CBF values than healthy controls in several brain regions, mainly in the limbic system and the default network (including the right precentral gyrus, the right parahippocampal gyrus, the bilateral superior parietal gyrus) (Table 2).

**TABLE 2:** Significant between‐group differences in CBF.

Parameter	AAL	Patients with ESRD	Healthy controls	*z* Value	*p* Value
PreCG.R	2	72.08 (57.1, 90.9)	58.31 (53.3, 66.6)	−2.663	.039
SMA.L	19	69.48 (59.7, 79.4)	58 (51.6, 63.7)	−3.27	.013
SMA.R	20	70.19 (59.1, 79.6)	58.36 (52.1, 64.3)	−3.41	.013
DCG.L	33	76.36 (62.4, 107.1)	65.80 (57.7, 71.3)	−2.607	.039
DCG.R	34	76.69 (61.7, 102.1)	62.31 (56.1, 73.9)	−2.635	.039
PHG.R	40	66.44 (49.6, 249.2)	50.95 (47.3, 60.7)	−2.663	.039
LING.L	47	83.73 (58.8, 279.6)	60.01 (57.8, 76.5)	−2.953	.023
LING.R	48	98.38 (58.6, 250.4)	60.50 (53.4, 80.1)	−2.635	.039
IOG.L	53	83.25 (61.9, 181.3)	59.32 (51.4, 64.5)	−3.644	<.001
IOG.R	54	78.91 (60.0, 150.2)	61.38 (53.5, 68.1)	−2.71	.039
FFG.L	55	72.26 (52.5, 218.7)	51.12 (46.7, 58.5)	−3.616	<.001
FFG.R	56	67.98 (54.2, 199.1)	51.4 (46.9, 59.3)	−3.504	<.001
SPG.L	59	73.21 (59.8, 109.6)	56.31 (57.9, 69.7)	−2.598	.039
SPG.R	60	67.82 (56.4, 88.1)	55.16 (58.5, 64.4)	−3.111	.02
IPL.L	61	74.73 (60.9, 74.7)	58.57 (54.2, 66.9)	−2.616	.039
IPL.R	62	76.41 (61.4, 98.6)	61.55 (55.5, 70.8)	−2.943	.023
PCL.L	69	66.99 (55.9, 76.5)	55.36 (51.6, 64.3)	−3.083	.02
PCL.R	70	74.87 (60.6, 84.3)	62.03 (53.9, 67.6)	−2.999	.023
TPOmid.R	88	68.77 (54.8, 168.2)	52.75 (50.4, 64.2)	−2.691	.039
ITG.L	89	74.90 (59.8, 182.1)	57.43 (50.6, 62.7)	−3.541	<.001
ITG.R	90	77.19 (60.9, 193.9)	58.65 (54.1, 66.7)	−3.392	.013

CBF data are expressed as median (lower and upper quartiles) /mL· (100 g·min) ^–1^.

Corrected using false‐discovery rate criterion and set at *p *< .05.

CBF, cerebral blood flow; AAL, automated anatomical labeling; ESRD, end‐stage renal disease; PreCG.R, right precental gyrus; SMA.L, left supplementary motor area; SMA.R, right supplementary motor area; DCG.L, left median cingulate and paracingulate gyri; DCG.R, right median cingulate and paracingulate gyri; PHG.R, right parahippocampal gyrus; LING.L, left lingual gyrus; LING.R, right lingual gyrus; IOG.L, left inferior occipital gyrus; IOG.R, right inferior occipital gyrus; FFG.L, left fusiform gyrus; FFG.R, right fusiform gyrus; SPG.L, left superior parietal gyrus; SPG.R, right superior parietal gyrus; IPL.L, left inferior parietal, but supramarginal and angular gyri; IPL.R, right inferior parietal, but supramarginal and angular gyri; PCL.L, left paracentral lobule; PCL.R, right paracentral lobule; TPOmid.R, right temporal pole: middle temporal gyrus; ITG.L, left inferior temporal gyrus; ITG.R, right inferior temporal gyrus.

After FDR correction, patients with ESRD were found to demonstrate significantly changed BC in 9 regions, Ne in 11 regions, and Dc in 9 regions (Table 3). The brain regions with increased nodal parameters mainly included the right superior frontal gyrus, orbital part and the right middle frontal gyrus, orbital part. The brain regions with decreased nodal parameters mainly included the bilateral middle frontal gyrus, and the right parahippocampal gyrus.

**TABLE 3:** Significant between‐group differences in topological parameters of functional networks.

Parameter	AAL	Patients with ESRD	Healthy controls	*t* Value	*p* Value
BC					
MFG.L	7	14.92 ± 6.04	18.37 ± 9.31	−2.022	.049
MFG.R	8	15.94 ± 5.59	19.78 ± 9.37	−2.294	.026
SMA.R	20	16.49 ± 7.99	12.21 ± 5.70	2.731	.01
ORBsupmed.L	25	14.08 ± 5.56	18.77 ± 9.28	−2.827	.01
PHG.R	40	14.08 ± 8.13	18.98 ± 8.63	−2.64	.01
PCL.R	70	13.81 ± 7.45	10.99 ± 5.04	2.028	.049
TPOsup.L	83	15.31 ± 6.27	20.49 ± 13.65	−2.268	.026
MTG.L	85	16.21 ± 6.75	21.62 ± 13.45	−2.361	.026
TPOmid.L	87	19.01 ± 7.26	22.89 ± 10.29	−1.998	.049
Ne					
PreCG.L	1	0.251 ± 0.014	0.243 ± 0.012	2.414	.033
ORBsup.R	6	0.255 ± 0.009	0.249 ± 0.012	2.579	.033
ORBmid.R	10	0.253 ± 0.012	0.245 ± 0.011	3.088	.01
SMA.L	19	0.243 ± 0.012	0.237 ± 0.010	2.229	.039
SMA.R	20	0.247 ± 0.012	0.241 ± 0.012	2.323	.039
PHG.R	40	0.241 ± 0.012	0.250 ± 0.013	−2.987	.01
SPG.L	59	0.244 ± 0.015	0.237 ± 0.013	2.051	.044
PCUN.L	67	0.239 ± 0.012	0.231 ± 0.012	2.767	.01
PCL.L	69	0.247 ± 0.013	0.239 ± 0.014	2.698	.01
PCL.R	70	0.245 ± 0.009	0.232 ± 0.015	4.666	<.001
ITG.R	90	0.246 ± 0.013	0.239 ± 0.016	2.108	.044
DC					
ORBsup.R	6	11.77 ± 1.66	10.96 ± 1.74	2.125	.046
ORBmid.R	10	11.41 ± 1.90	10.36 ± 1.50	2.722	.01
PCG.L	35	9.92 ± 1.47	10.77 ± 1.76	−2.358	.031
PCG.R	36	9.41 ± 1.48	10.13 ± 1.69	−2.206	.046
PHG.R	40	9.71 ± 1.8	11.25 ± 1.9	−3.714	<.001
PCUN.L	67	9.35 ± 1.56	8.56 ± 1.56	2.281	.031
PCL.R	70	10.23 ± 1.21	8.83 ± 2.29	3.496	<.001
HES.L	79	10.51 ± 1.89	11.62 ± 1.52	−2.857	.01
TPOsup.L	83	10.9 ± 2.15	11.92 ± 1.97	−2.196	.046

Data are expressed as mean ± standard deviation. Corrected using false‐discovery rate criterion and set at *p* < .05.

AAL, automated anatomical labeling; ESRD, end‐stage renal disease; BC, betweenness centrality; Ne, node efficiency; DC, degree centrality; MFG.L, left middle frontal gyrus; MFG.R, right middle frontal gyrus; SMA.R, right supplementary motor area; ORBsupmed.L, left superior frontal gyrus, medial orbital; PHG.R, right parahippocampal gyrus; PCL.R, right paracentral lobule; TPOsup.L, left temporal pole: superior temporal gyrus; MTG.L, left middle temporal gyrus; TPOmid.L, left temporal pole: middle temporal gyrus; PreCG.L, left precental gyrus; ORBsup.R, right superior frontal gyrus, orbital part; ORBmid.R, right middle frontal gyrus, orbital part; SMA.L, left supplementary motor area; SPG.L, left superior parietal gyrus; PCUN.L, left precuneus; PCL.L, left paracentral lobule; ITG.R, right inferior temporal gyrus; PCG.L, left posterior cingulate gyrus; PCG.R, right posterior cingulate gyrus; HES.L, left Heschl's gyrus.

### 3.3 Changes in NVC

#### 3.3.1 Changes in NVC at the level of the whole brain

**FIGURE 2:**
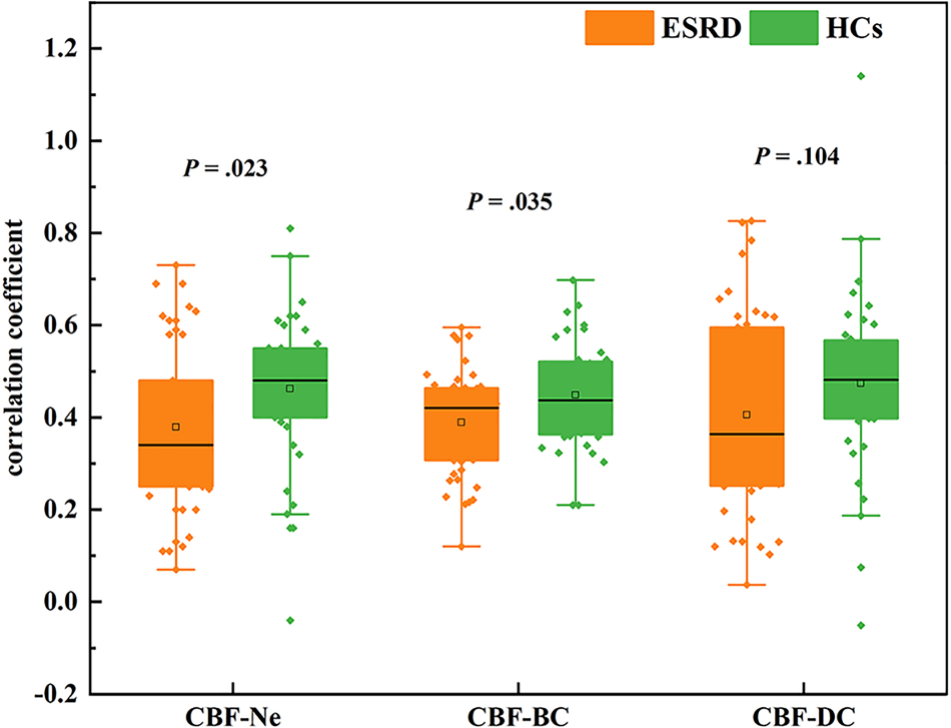
Differences in the overall CBF‐Ne, CBF‐BC, and CBF‐DC coupling coefficients at the whole brain level between the two groups. *p* < .05 indicates a significant difference between groups. The midline represents the median. The box boundaries represent the 25th and 75th quartiles. The upper and lower lines are the upper and lower limits, which are the maximum and minimum values within the nonanomaly range, respectively. Points exceeding the upper and lower limits represent outliers.

All patients and healthy controls showed a significant correlation between CBF and nodal parameters. Interestingly, the overall CBF‐Ne and CBF‐BC coupling coefficients were significantly lower in patients than in healthy controls (CBF‐Ne coefficient: rESRD = 0.34 (0.25, 0.53), rHC = 0.48 (0.40, 0.55), *p *= .023, CBF‐BC coefficient: rESRD = 0.42 (0.31, 0.47), rHC = 0.44 (0.36, 0.52), *p *= .035). No significant differences were found between these two groups in CBF‐DC coefficient (rESRD = 0.36 (0.25, 0.60), rHC = 0.48 (0.40, 0.57), *p* = .104). Coefficient data are expressed as median (lower and upper quartiles) (Figure 2).

#### 3.3.2 Changes in NVC at the level of the local brain region

**FIGURE 3:**
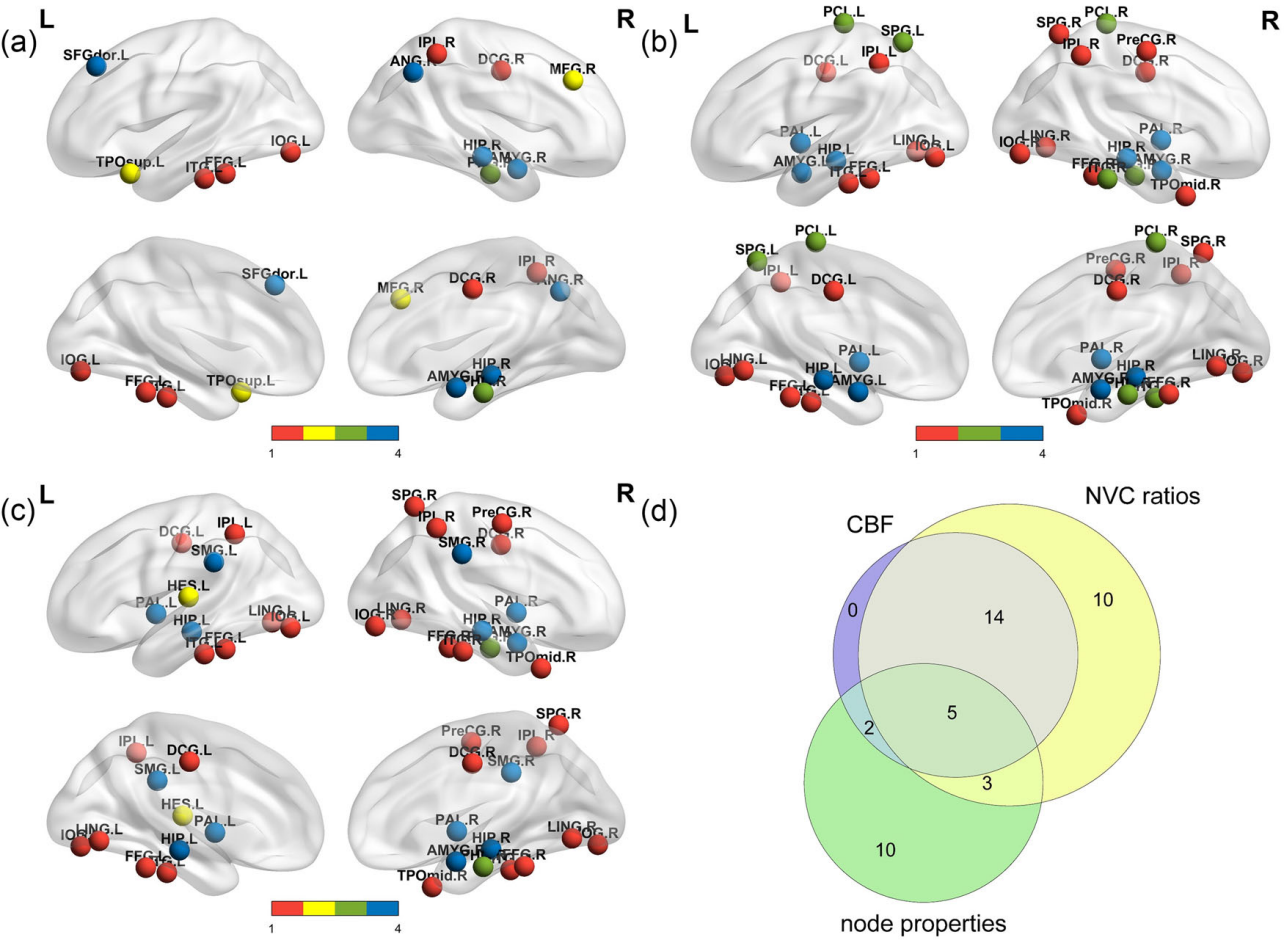
(a–c) Brain regions with significant between‐group differences in CBF/ BC, CBF/ Ne, and CBF/ DC coupling ratios. Red nodes represent ratio changes accompanied by CBF changes only. Yellow nodes represent ratio changes accompanied by node property changes only. Green nodes represent ratio changes accompanied by both CBF and node property changes. Blue nodes represent ratio changes only. (**d**) Differential brain region overlap map for coupling ratios, CBF and node properties. The counts in the figure represent the number of abnormal brain regions.. PreCG.R, right precental gyrus; DCG.L, left median cingulate and paracingulate gyri; DCG.R, right median cingulate and paracingulate gyri; LING.L, left lingual gyrus; LING.R, right lingual gyrus; IOG.L, left inferior occipital gyrus; IOG.R, right inferior occipital gyrus; FFG.L, left fusiform gyrus; FFG.R, right fusiform gyrus; SPG.R, right superior parietal gyrus; IPL.L, left inferior parietal, but supramarginal and angular gyri; IPL.R, right inferior parietal, but supramarginal and angular gyri; TPOmid.R, right temporal pole: middle temporal gyrus; ITG.L, left inferior temporal gyrus; ITG.R, right inferior temporal gyrus; MFG.R, right middle frontal gyrus; HES.L, left Heschl's gyrus; TPOsup.L, temporal pole: superior temporal gyrus; PHG.R, right parahippocampal gyrus; SPG.L, left superior parietal gyrus; PCL.L, left paracentral lobule; PCL.R, right paracentral lobule; SFGdor.L, left superior frontal gyrus, dorsolateral; HIP.L, left hippocampus; HIP.R, right hippocampus; AMYG.L, left amygdala; AMYG.R, right amygdala; SMG.L, left supramarginal gyrus; SMG.R, right supramarginal gyrus; ANG.R, right angular gyrus; PAL.L, left lenticular nucleus, pallidum; PAL.R, right lenticular nucleus, pallidum.

After FDR correction, compared with healthy controls, patients with ESRD demonstrated an increase in CBF/nodal property ratio in various brain regions. These changes fell into 1 of 4 categories. The first category included coupling ratio changes accompanied by changes in CBF only; these mainly involved the right precentral gyrus, the bilateral medial and paracentral cingulate gyrus, the bilateral lingual gyrus, the bilateral inferior occipital gyrus, and the bilateral fusiform gyrus. The second category included coupling ratio changes accompanied by changes in node properties only, mainly involving the right middle frontal gyrus and the left Heschl's gyrus. The third category included coupling ratio changes accompanied by changes in both CBF and nodal properties; these changes mainly involved the right parahippocampal gyrus and the bilateral paracentral lobule. The last category included coupling ratio changes only, which were found in the bilateral hippocampus, the bilateral amygdala, and the left superior frontal gyrus, dorsolateral (Figure 3).

### 3.4 Correlation analysis

**FIGURE 4:**
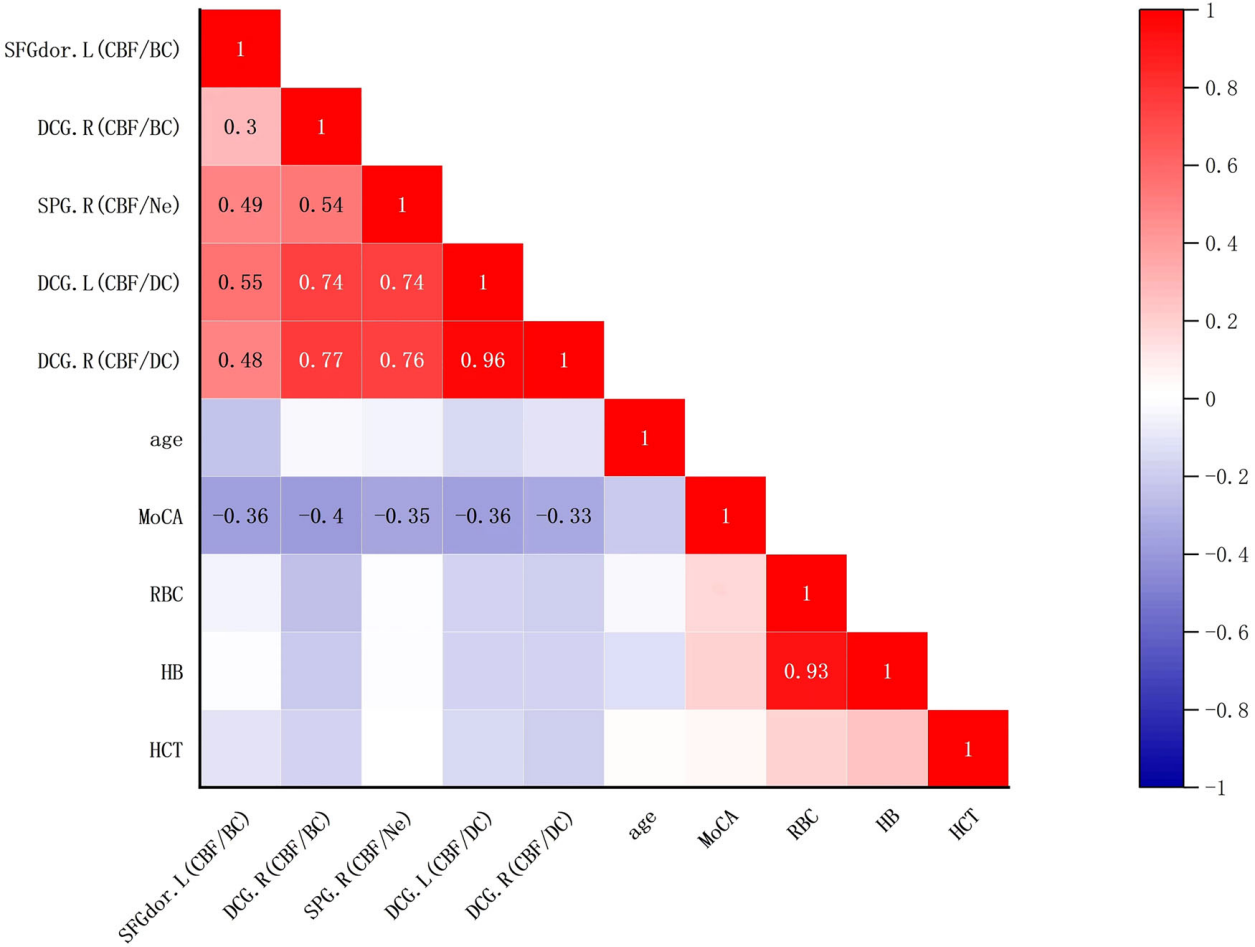
Correlation between coupling ratio parameters, MoCA scores and clinical indicators in patients with ESRD. The color scale from blue to red represents the *r* value from −1 to 1. *p < .05. The values of the correlation coefficients marked in the graph are all statistically significant. RBC, red blood cell count; HB, hemoglobin; Hct, hematocrit. SFGdor.L, left dorsolateral superior frontal gyrus; DCG.R, right median cingulate and paracingulate gyri; SPG.R, right superior parietal gyrus; DCG.L, left median cingulate and paracingulate gyri.

Among patients with ESRD, there was a negative correlation between CBF/BC ratio and MoCA score in the left dorsolateral superior frontal gyrus (*r* = −0.361, *p* = .015) and the right median cingulate and paracingulate gyri (*r* = −0.396, *p* = .007); a negative correlation between CBF/Ne ratio and MoCA score in the right superior parietal gyrus (*r* = −0.345, *p* = .020); and a negative correlation between CBF/DC ratio and MoCA score in the bilateral median cingulate and paracingulate gyri (left: *r* = −0.364, *p* = .012; right: *r* = −0.334, *p* = .024). None of the CBF‐node property coupling coefficients were correlated with cognitive scores. Given that anemia may be a predominant risk factor for neurocognitive impairment, the five previously mentioned correlations still remained even after adjusting for the hemoglobin and hematocrit levels (*r* = −0.367, *p* = .016; *r* = −0.372, *p* = .014; *r* = −0.340, *p* = .026; *r* = −0.362, *p* = .017; *r* = −0.324, *p* = .034). The correlation between the increased CBF/DC ratio of the right superior parietal gyrus and poorer MoCA scores was statistically marginally significant after adjusting for the hemoglobin and hematocrit levels (*r* = −0.303, *p *= .048). No significant correlations between the increased CBF/DC ratio of the superior parietal gyrus and the poorer MoCA scores were found before adjusting for the hemoglobin and hematocrit levels (*r* = −0.283, *p* = .059). All correlation *p*‐values were uncorrected (Figure 4).

## 4 DISCUSSION

This study demonstrated that patients with ESRD who are undergoing dialysis are more likely than healthy controls to demonstrate abnormal NVC. In our study, patients with ESRD showed significantly decreased global coupling coefficients and increased regional coupling ratios compared with HC, mainly in the limbic system and default network. In patients, negative correlations were seen between coupling ratios and MoCA scores in many brain regions, and the correlations still remained even after adjusting for the hemoglobin and hematocrit levels. Our findings supported that cognitive impairment in these patients may have a neurovascular cause. There are two main hypotheses regarding the development of cognitive dysfunction in patients with ESRD, namely the vascular hypothesis and the neurodegenerative hypothesis (Arnold et al., 2016; Qian et al., 2021). These two hypotheses would cause an increase in cerebral perfusion and a decrease in neuronal activity, respectively. Our research also supported these two hypotheses.

In this study, a correlation was observed between CBF and the nodal parameters BC, Ne, and DC in all study participants, supporting the tight NVC in the normal brain. BC, Ne, and DC reflect the influence on the network circulation or connectivity, the transmission capacity of nodes, and the importance of individual nodes in the network, respectively. Therefore, the NVC patterns reflect the coordination between blood supply and brain connectivity in various aspects. In our study, patients with ESRD showed significantly decreased global CBF‐Ne and CBF‐BC couplings compared with HC. In general, higher degree of brain connectivity indicates more active metabolism and may require more energy consumption. The imbalance between brain connectivity and metabolic supply may implicate a decline in brain functioning in ESRD, presumably resulting in overall neurovascular decoupling in patients. There are several possible explanations for this finding. NVC depends on the integrity of the neurovascular unit (neurons, glial cells, and vascular components). A large accumulation of uremic toxins in the neurovascular unit among patients with ESRD will produce neurotoxicity on neuronal cells and accelerate their degeneration and modulation (Faucher et al., 2023). At this time brain connectivity declines along with decreased neural activity. Uremic toxins can also damage neurons through neuroinflammation and oxidative stress on glial cells (Li et al., 2021). In addition, total homocysteine levels are high in patients with ESRD, potentially leading to endothelial dysfunction, atherosclerosis, and parenchymal ischemia (Paganelli et al., 2021). Previous research has shown that the endothelial and nitric oxide signaling pathways play a key role in NVC‐related vascular regulation (Rosengarten et al., 2003). It is also possible that they were anemic based on their hemoglobin and hematocrit values. Given that hemoglobin and hematocrit are known to be factors in determining CBF, an increase in CBF does not always equate to an increase in the BOLD signal. CBF itself can rise. The correlation coefficient can decline as a result. Anemia plays a substantial role in the decline of NVC in dialysis patients. Unfortunately, we found no significant correlation between any of the three coupling coefficients and MoCA scores, which may be attributed to the high subjective nature of MoCA scores. Of note, no between‐group differences were observed for CBF‐DC coupling coefficients. Because DC reflects connections between brain regions, whereas BC and Ne reflect the ability to transmit information between brain regions, we speculate that dialysis may have a more pronounced effect on transmission efficiency and a weaker effect on the number of connections between brain regions.

Even in the setting of normal NVC, since the coupling ratio just represents the balance between CBF and brain connectivity, the coupling ratio may alter for a variety of reasons. To determine the cause of the shift in coupling rate, we looked at how CBF and node characteristics changed in each brain region separately. Following analysis of all brain areas with different coupling ratios between HC and ESRD patients, changes in CBF and nodal parameters of these brain areas were noted. In this study, we observed 4 types of increased coupling ratio changes in dialysis patients compared to healthy controls. The first type was accompanied by changes in CBF only. It may be that changes in CBF precede changes in brain function, resulting in NVC discordance, suggesting that we need to prevent decreased NVC by intervening in brain perfusion early before brain function is affected in these brain regions. This increase in CBF among patients with ESRD could be related to a number of factors. First, patients undergoing hemodialysis have an increased risk of developing uremic anemia (Zheng et al., 2016). The decreased blood viscosity and decreased oxygen delivery associated with uremic anemia have been found to increase CBF and lead to adverse cerebrovascular effects. Patients with ESRD are also at risk of chronic arterial hypertension (Mailloux & Haley, 1998), and prolonged hypertension can lead to endothelial dysfunction and microangiopathy. Both of these factors can lead to disruption of the blood‐brain barrier (BBB), which an animal model study found could result in cerebral hyperperfusion (Sakaki et al., 1992) Dialysis patients affect CBF in these above structural and metabolic aspects. It also suggests that we should intervene CBF from the above aspects to prevent neurovascular decoupling. In our study, as in previous studies, the brain regions demonstrating increased CBF were mainly in the limbic system (including the median cingulate and paracingulate gyri), frontal lobes, and temporal lobes (Cheng et al., 2019). The limbic system, which is interconnected through the Papez loop and has extensive connections with other brain structures (e.g., the neocortex, thalamus, and brainstem) and enables information exchange between the midbrain, mesencephalon, and neocortical structures (Papez, 1995; Roxo et al., 2011) Patients in this study also demonstrated a negative correlation between the coupling ratio indexes of the median cingulate and paracingulate gyri, the superior parietal gyrus and MoCA scores. More strongly speculating that this imbalance in brain function and perfusion changes resulting in NVC dysfunction, leading to cognitive decline. Increased coupling ratios were also observed in the bilateral lingual gyrus, inferior occipital gyrus, and fusiform gyrus among patients with ESRD. In a previous study on semantic memory (Chen et al., 2020), researchers discovered that nodal centrality values in the left fusiform gyrus were significantly linked to overall semantic processing performance in patients with semantic dementia. This suggests that the left fusiform gyrus is an important semantic center, and it has white matter connections to nine brain regions, such as the left lingual gyrus and inferior occipital gyrus. The patients in our study performed poorly on the memory portions of the MoCA, perhaps because of NVC deficits in these brain regions.

The second type of coupling ratio differences was accompanied by changes in nodal properties only. These brain regions have lower nodal properties, suggesting that the brain connectivity in these brain areas is more vulnerable to damage and that changes in NVC are mainly mediated by decreased brain connectivity. Possible causes of this altered brain connectivity include decreased clearance of metabolites and disruption of the Na+‐K+ pump (Bugnicourt et al., 2013; Kaji & Thomas, 1987; Xie et al., 2022). The right middle frontal gyrus, an important component of the dorsolateral prefrontal cortex (DLPFC), is commonly associated with executive functions (including selective attention and verbal working memory) (Curtis & D'Esposito, 2003). Previous research using rs‐fMRI and ALFF‐based analyses demonstrated reduced spontaneous neural activity in the DLPFC of patients undergoing dialysis, and this reduced activity was associated with short‐term verbal memory deficits (Li et al., 2018). Although we did not detect a correlation between the coupling index in this brain region and MoCA score, we cannot deny the contribution of NVC status in this brain region to cognitive function. Thus, it is possible that noninvasive neural interventions targeting specific brain regions could be adopted to effectively inhibit cognitive impairment and improve cognitive potential in ESRD patients. In addition, we found the phenomenon of nodal properties rising in some brain regions, but the coupling ratio did not change. Previous research has also shown that clinical depression is prevalent in patients undergoing hemodialysis and that functional connectivity of the amygdala‐prefrontal‐posterior cingulate gyrus‐limbic circuits is impaired in depressed patients undergoing hemodialysis (Chen et al., 2017). Our findings may offer evidence of a compensatory mechanism for increased Ne and BC in the right superior frontal gyrus, orbital part and the right middle frontal gyrus, orbital part due to marked impairment of the limbic system. Although no differences in coupling ratios were observed in these brain regions, as ESRD progresses, overcompensation may cause an imbalance in NVC. Unfortunately, we did not assess the emotional state of patients in this study; future studies should evaluate this factor.

The third type of coupling ratio differences was accompanied by changes in both CBF and nodal properties. All three imaging indices of these brain regions were altered, suggesting that these brain regions are more sensitive to all aspects of damage. Importantly, we found that the right parahippocampal gyrus (PHG) demonstrated this variation. The PHG is a crucial component of the mesial temporal lobe (MTL), which includes the hippocampus and its surrounding cortical structures. The PHG plays a critical role in transmitting temporal and spatial information to the hippocampus, and is responsible for integrating information, thereby connecting it to the wider memory network of the brain (Sharma et al., 2021). Previous research has shown that the MTL plays an important role in the encoding and consolidation of situational memory (King‐Stephens et al., 2015). In our study, patients demonstrated poorer results than healthy controls on the memory portion of the MoCA, presumably because the PHG is susceptible to changes in NVC.

The last type of coupling ratio change involved changes in the ratios alone. These regions may be so because the fact that local brain areas are not activated, no abnormality is detected in the brain connectivity index. However, the coupling ratio index can now be used to determine whether there is a coupling imbalance. This ratio observation perspective served to amplify intergroup differences, identifying abnormal regions not detected by CBF or nodal properties analyses. CBF, nodal properties, and CBF/nodal properties ratios may provide complementary information and ratio results are more sensitive. They should be used jointly to explore brain studies in patients with ESRD, and to prevent underdiagnosis of some brain regions. We have screened out regions such as the bilateral hippocampus, the bilateral amygdala and the left superior frontal gyrus, dorsolateral. The dorsolateral superior frontal gyrus is a key node in the dorsal attentional network and is involved in the basic cognitive selection of sensory information and responses, and also in the correlation analysis we found that the coupling ratio index in it was negatively correlated with MoCA scores. In a previous study of patients with depressive mood disorders, β waves were observed in the amygdala and hippocampus, two deep brain regions associated with memory and negative emotions (Kirkby et al., 2018). We should focus on the study of patients' emotions in the future. One of the most fascinating features of the hippocampus is its extraordinary capability for adult neurogenesis. This brain region can continuously generate new neurons that functionally integrate into existing neural circuits and effectively contribute to complex behaviors (Gonçalves, Schafer, & Gage, 2016). Thus, an abnormal coupling ratio in the bilateral hippocampus may be associated with a disruption in neurogenesis, which in turn leads to a decrease in NVC.

In our study, we found that the increased coupling ratio in the left dorsolateral superior frontal gyrus, the right median cingulate and paracingulate gyri, the right superior parietal gyrus, and the left median cingulate and paracingulate gyri was correlated with the poorer MoCA scores. However, it is rather difficult to uncover the causes of neurocognitive dysfunction in this study. Because patients exhibit decreased hemoglobin and hematocrit due to the anemia that occurs secondary to renal failure. Although no correlation was found between hemoglobin, hematocrit, and MoCA scores, decreased hematocrit can lead to decreased brain oxygen delivery, with a detrimental effect on brain metabolism (Pereira et al., 2005). It is possible that decreased hematocrit caused low oxygen delivery and altered NVC, contributing to cognitive dysfunction. After adjusting for the hemoglobin and hematocrit levels, interestingly these correlations still remained, and even a new brain region with correlations with MoCA was identified: the right superior parietal gyrus, indicating that when the effect of anemia was excluded, abnormal NVC indexes remained correlated with neurocognitive impairment. Our finding was different from that reported in the study by Jiang et al. (Jiang et al., 2016). In that study, the authors found that increased CBF of multiple cerebral regions was correlated with neuropsychological tests in ESRD patients; however, the correlations were absent or shrank after adjusting for the hemoglobin levels. There may be several reasons for the different results. In the study by Jiang et al. (2016), the patients included ESRD patients without dialysis, ESRD patients with peritoneal dialysis, and ESRD patients with hemodialysis. However, the patients in our study only included the ESRD patients with hemodialysis. The different dialysis modalities and whether having dialysis therapy may have a different effect on the blood system, such as the changes of CBF and the degree of anemia, which caused the different findings between Jiang et al.’s study and ours. There is increasing evidence that the HD procedure itself might contribute to brain injury (Hsieh et al., 2009; Murray et al., 2013; Polinder‐Bos et al., 2020; Polinder‐Bos et al., 2018; Zhang et al., 2015), which may cause further neurocognitive impairment. It is also possible that due to effective erythropoietin therapy in our patient, the hemoglobin and hematocrit were slightly lower than normal to the point that the cognitive effects were less severe. Taken together, these findings suggest that cognitive impairment in patients undergoing dialysis may be related to dialysis‐induced abnormal NVC in certain brain regions. NVC dysfunction in these ESRD‐susceptible brain regions may reflect patterns of brain function impairment.

## 5 LIMITATIONS

First, the NVC parameters assessed in this study were less correlated with MoCA scores; we cannot exclude that this is due to the subjective nature of MoCA scores. In future studies, we will use a more objective scale to assess the cognitive function of patients. Second, CBF and nodal properties indirectly reflect vascular response and brain connectivity, respectively. Therefore, correlations and ratios were analyzed as indirect measures of NVC in patients. This technical limitation may have affected our analysis of NVC in patients. In future studies, we intend to fuse the 2 MRI techniques to explore more direct NVC parameters. Third, this study lacked further follow‐up studies. Future studies should focus on longitudinal interpretation of dynamic changes in cognitive impairment during long‐term dialysis and potential mechanisms of NVC in patients with ESRD.

## 6 CONCLUSION

This study demonstrated that in ESRD patients receiving dialysis, NVC anomalies may be connected to the emergence of cognitive impairment. These findings provide multiparameter neuroimaging evidence regarding the effect of NVC on cognitive function in patients undergoing dialysis and suggest that dialysis‐related neurovascular biomarkers could be used to monitor the progression of cognitive impairment in this patient population.

## AUTHOR CONTRIBUTIONS

Conceptualization: Wei Sun and Haifeng Shi; methodology: Chen Li and Haifeng Shi; software: Wei Sun, Chen Li, and Zhuqing Jiao; data curation: Tongqiang Liu; investigation: Wei Sun, Chen Li, and Haifeng Shi; validation: Wei Sun and Haifeng Shi; formal analysis: Wei Sun and Zhuqing Jiao; supervision: Tongqiang Liu and Haifeng Shi; funding acquisition: Zhuqing Jiao and Haifeng Shi; visualization: Wei Sun, Chen Li, and Haifeng Shi; project administration: Wei Sun; resources: Chen Li, Tongqiang Liu, and Haifeng Shi; writing—original draft: Wei Sun and Chen Li; writing—review & editing: Wei Sun and Haifeng Shi.

## FUNDING

This work was supported by Changzhou Sci&Tech Program (CE20235062), Clinical Research Project of Changzhou Medical Center of Nanjing Medical University (CMCC202306) and Top Talent of Changzhou “The 14th Five‐Year Plan” High‐Level Health Talents Training Project (2022CZBJ072).

## CONFLICT OF INTEREST STATEMENT

The author declares that the research was conducted in the absence of any commercial or financial relationships that could be construed as a potential conflict of interest.

### PEER REVIEW

The peer review history for this article is available at https://publons.com/publon/10.1002/brb3.3598.

## Data Availability

The data that support the findings of this study are available on request from the corresponding author. The data are not publicly available due to privacy or ethical restrictions.
